# Modeling and Analysis of a SiC Microstructure-Based Capacitive Micro-Accelerometer

**DOI:** 10.3390/ma14206222

**Published:** 2021-10-19

**Authors:** Xiang Tian, Wei Sheng, Zhanshe Guo, Weiwei Xing, Runze Tang

**Affiliations:** 1 School of Instrumentation and Optoelectronic Engineering, Beihang University, Beijing 100191, China; guozhanshe@buaa.edu.cn (Z.G.); xingweiwei@buaa.edu.cn (W.X.); 2 School of Energy and Power Engineering, Beihang University, Beijing 100191, China; tangrunze@buaa.edu.cn

**Keywords:** MEMS (microelectromechanical system) sensors, accelerometer, silicon carbide, modal analysis, dynamic characteristics, frequency characteristics

## Abstract

In this study, a comb-type capacitive accelerometer based on a silicon carbide (SiC) microstructure is presented and investigated by the finite element method (FEM). It has the advantages of low weight, small volume, and low cross-coupling. Compared with silicon(111) accelerometers with the same structure, it has a higher natural frequency. When the accelerometer vibrates, its resistive force consists of two main components: a viscous damping and an elastic damping force. It was found that viscous damping dominates at low frequency, and elastic damping dominates at high frequency. The second-order linear system of the accelerometer was analyzed in the time-frequency domain, and its dynamic characteristics were best when the gap between the capacitive plates was 1.23 μm. The range of this accelerometer was 0–100 g, which is 1.64 times that of a silicon(111) accelerometer with the same structure. In addition, the accelerometer could work normally at temperatures of up to 1200 °C, which is much higher than the working temperatures of silicon devices. Therefore, the proposed accelerometer showed superior performance compared to conventional silicon-based sensors for inertial measurements.

## 1. Introduction

In the early 1940s, German researchers developed the world’s first pendulous gyroscope accelerometer [1]. It was applied in the V-2 rocket, which greatly improved the rocket’s hit rate [2]. For more than half a century thereafter, due to the demand for inertial measurement units (IMUs) in aviation, navigation, and aerospace, various types of accelerometers emerged, and their performance and accuracies have been greatly improved [3,4].

In the 1980s, micro-accelerometers were made using integrated circuits (ICs) and micromachining, which gave them the advantages of small volume [5], light weight [6], low power consumption [7], low cost [8], easy integration [9], strong overload capacity [10], and mass production [11]. These not only became the core components of micro-inertial measurement units (MIMUs) [12] but also rapidly expanded into other civil fields [13,14]. In 1979, Roylance and Angell from Stanford University produced the first open-loop silicon accelerometer using silicon ICs [15] and released the product in the early 1980s. In the late 1980s, people began to study various closed-loop force-balanced silicon micro-accelerometers and made great progress. In 1989, Analog Devices Inc. (ADI) began research into silicon comb-type capacitive micro-accelerometers [16], and cooperated with the Siemens company to develop its electronic measurement circuit in 1990. In 1992, the accelerometer developed by ADI met the performance index requirements for automobile safety airbags. It was put into production in 1993, and a series of products followed: ADXL50 [17], ADXL05 [18], and others [19,20].

In 1995, the electronic circuit of the accelerometer, developed by the LITEF company in Germany, adopted the scheme of pulse-width modulation, digital output, and closed-loop control. The range of the accelerometer was ±10 g; the scale factor stability was 300 ppm, and the bias stability was 250 μg. This kind of micro-accelerometer was combined with a fiber optic gyroscope (FOG) to form the MIMU for vehicle navigation [21]. The Applied MEMS company in the United States developed a very low-noise application specific integrated circuit (ASIC) micromachined accelerometer for seismic exploration in 2002. This single-axis accelerometer used the ASIC for the force feedback system, and it consisted of two parts: a capacitance detector and a closed-loop force feedback circuit. The noise could be kept below −150 dB g/Hz^1/2^ in the frequency range of 3–200 Hz.

According to the detection principle, micro-accelerometers can be divided into piezoresistive [22], piezoelectric [23], resonant [24], capacitive [25], and other forms. Among them, capacitive accelerometers have the advantages of high accuracy [26], low-noise [27], low temperature sensitivity [28], low power consumption [29], and a simple structure [30]. Therefore, they gradually became mainstream in the development of micro-silicon acceleration sensors.

Over the past few decades, silicon devices have significantly improved. However, they are approaching the performance limits defined by the basic material properties of silicon [31], and further performance improvements can only be achieved by migrating to more powerful semiconductor materials [32]. As a representative of the third generation, silicon carbide has excellent physical, chemical, and electrical properties, such as a wide band-gap [33], high mechanical strength [34], high thermal conductivity [35], high melting point [36], and corrosion resistance [37]. Compared with the Si devices, SiC MEMS devices can work at higher temperatures and in harsher environments [38]. The wide band-gap and high thermal stability of SiC allow certain types of devices to operate indefinitely at junction temperatures of 300 °C or higher without measurable performance degradation. The potential markets for SiC sensors include the Radio Frequency (RF) MEMS [39], the pressure sensors used in the petroleum industry [40], the acceleration sensors in aircraft engines and motors [41], and the optical MEMS [42].

In this paper, we propose a differential capacitive accelerometer based on a SiC microstructure by FEM. Firstly, the working principle and the process flow of the accelerometer was introduced. Then the structural parameters of the accelerometer were optimized to avoid cross-coupling. In the modal analysis, we found that a SiC-based accelerometer had a higher natural frequency compared to a Si-based accelerometer with the same structure. Secondly, a good damping design can improve the dynamic characteristics [43]. Therefore, the squeeze film air damping of the accelerometer was studied, and we found that, when the vibration frequency was low, the viscous damping force of the air film increased linearly with an increase in the vibration frequency, and the elastic damping force increased slowly. However, when the vibration frequency was high, the viscous damping force of the air film decreased with an increase in vibration frequency, and the elastic damping force increased rapidly. Moreover, the accelerometer’s second-order linear system was analyzed in the frequency and time domains. The analysis showed that the dynamic characteristics were at their best when the gap between the capacitive plates was 1.23 μm. That is, the accelerometer has a large bandwidth and no resonant peak in its amplitude-frequency curve, and a fast output in its step response. Lastly, the performance characteristics of the SiC-based accelerometer showed a range of 0–100 g, which is 1.64 times of that of a silicon(111) accelerometer of the same structure. In addition, the SiC-based accelerometer worked normally at high temperatures up to 1200 °C, which is much higher than the working temperature (250 °C) of the silicon devices. In summary, the proposed accelerometer showed great potential as an alternative to conventional Si sensors in inertial measurement applications.

## 2. Working Principles

### 2.1. Design of a Comb-Type Capacitive Accelerometer

**Figure 1:**
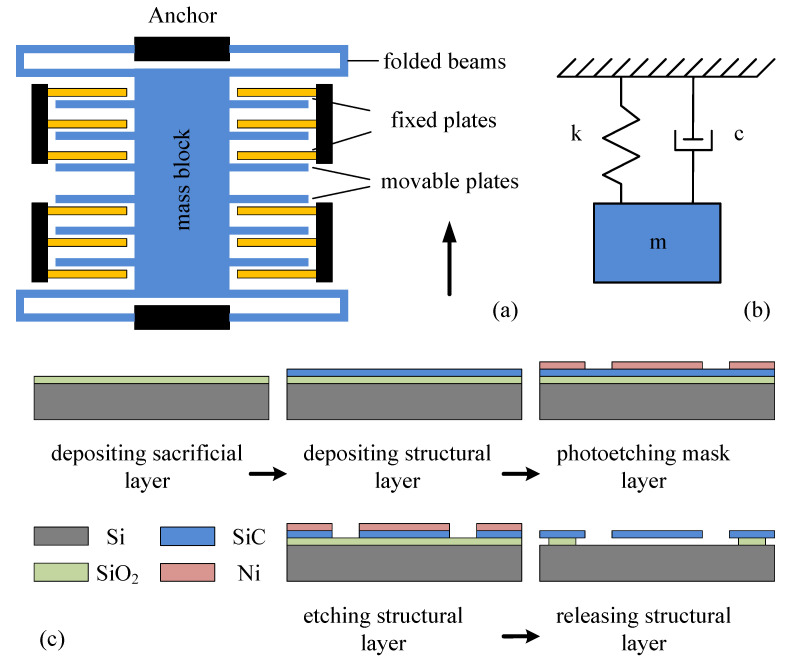
Model of a single-axis, comb-type capacitive micro accelerometer. (**a**) Physical model of the accelerometer, (**b**) mechanical model of the accelerometer, (**c**) process flow of the accelerometer.

Figure 1a shows the structural model of a single-axis, comb-type capacitive micro-accelerometer consisting mainly of a sensitive mass block with comb fingers, fixed electrode plates, and two pairs of folded support beams. The middle part is the mass block, which is fixed on the substrate by four thin beams. The mass block can move freely along the direction perpendicular to the thin beams. The comb fingers extend from both sides of mass block, and each comb finger is a movable electrode plate of the variable capacitor. The fixed electrode plates and the movable electrode plates are arranged alternately, and they form differential detection electrodes.

When the system is accelerated, the mass block is subjected to inertial force, and has a certain displacement in the acceleration–detecting direction. Then, the displacement is converted into capacitance changes on the capacitive plates, and the acceleration value can be obtained by measuring the capacitance changes.

Figure 1c shows the process flow of the accelerometer. First, a low-temperature oxidation (LTO) sacrificial layer is deposited on the silicon substrate, which not only releases the structure, but also ensures the insulation between the electromechanical structure and the substrate. Second, silicon carbide is deposited as a structural layer by low-pressure chemical vapor deposition (LPCVD) technique. Third, the nickel mask layer is formed by the stripping process for the next etching process. Fourth, the structural layer is processed by inductively coupled plasma (ICP) etching. Finally, the wafer is placed in an HF solution for wet etching to release the structure.

### 2.2. Modeling of the Capacitive Accelerometer

According to Newton’s second law, this kind of micromachined accelerometer can be equivalent to a second-order mass-spring-damping model, as shown in Figure 1b. Its differential equation is shown below [44]:

(1) $$m\cdot \frac{d^{2}x(t)}{dt^{2}}+c\cdot \frac{dx(t)}{dt}+k\cdot x(t)=m\cdot a(t)$$

where m is the mass of the sensitive mass block; c is the viscous damping coefficient of the system; k is the stiffness coefficient of the system; x(t) is the displacement of the mass block; a(t) is the acceleration of the measured object; and t is time.

In addition, the natural frequency of the accelerometer is $\omega_{n}=\sqrt{\frac{k}{m}}$ and the relative damping ratio of the accelerometer is $\varsigma =\frac{c}{2\sqrt{mk}}$.

### 2.3. Parameter Setting of the Capacitive Accelerometer

It is relatively easy to calculate the mass of the micro-accelerometer. Assuming that the density of the material is ρ, the surface area of the structure is s, and the thickness of the structure is T, the mass of the whole structure is

(2) m=ρsT

**Figure 2:**
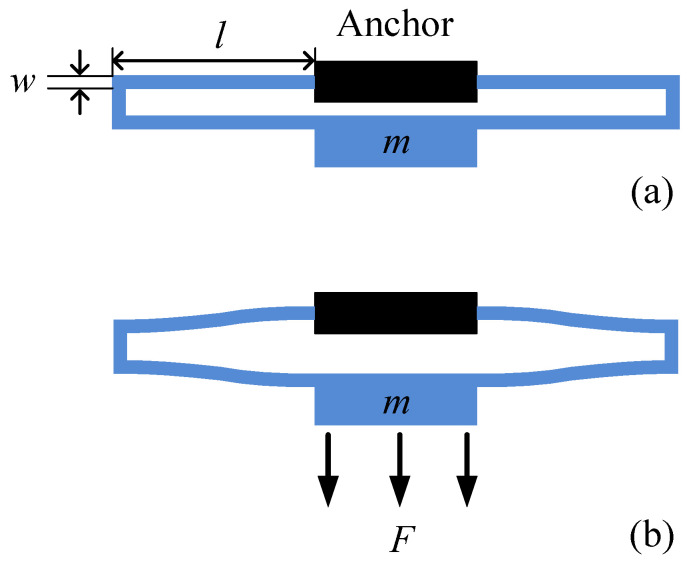
Structural diagram of a pair of folded support beams, in which m represents the mass of the sensitive mass block. (**a**) Dimensions of folded support beams; (**b**) deformation diagram of the folded support beams.

The stiffness of the micro-accelerometer is provided by four folded beams, the length and width of which are l and w, respectively, as shown in Figure 2a. When the micro-accelerometer is moved upwards, the mass block is subjected to a downward inertial force F. The deformation diagram of a pair of folded support beams is shown in Figure 2b.

According to the principle of material mechanics, the stiffness coefficient of the micro-accelerometer can be described as [45]

(3) $$k=\frac{2Ew^{3}T}{l^{3}}$$

where E is the elastic modulus of the material.

**Figure 3:**
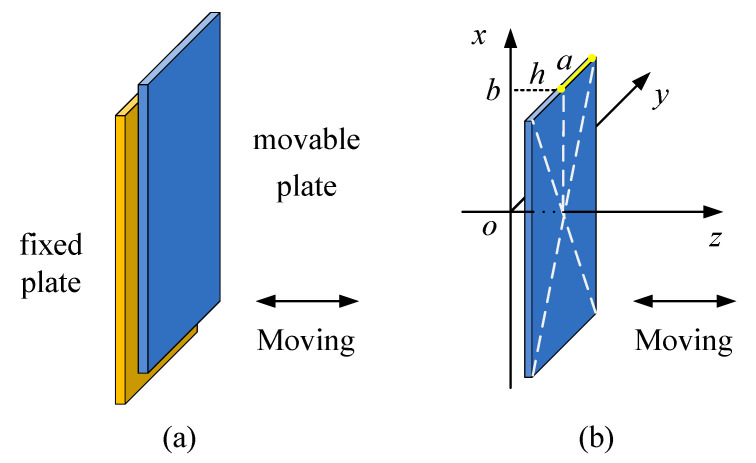
(**a**) Squeeze-film air damping between the movable electrode plate and fixed electrode plate in the capacitive accelerometer, (**b**) schematic diagram of squeeze-film air damping.

The damping of the MEMS accelerometer usually includes structural damping and air damping between moving parts. Under normal working conditions, structural damping is much lower than air damping, so it can be ignored. Air damping can be divided into squeeze-film air damping and slide-film air damping. For the capacitive accelerometer in this article, when it is accelerated, due to the existence of inertial force, the mass block with movable electrode plates undergoes a small displacement, resulting in capacitance changes. At this moment, the movable electrode plates move towards the fixed electrode plates, as shown in Figure 3a, and the air in the gaps between them is squeezed. The air acts as a damper, and it hinders the motion of the moving plates. Additionally, the movable plates may move away from the fixed plates, and the air between them also hinders the movements of the moving plates. This kind of damping is called squeeze-film air damping. In this process, the force that hinders the motion of the plate due to the viscous effect is the viscous damping force. However, when the vibration frequency of a movable plate is higher, there is not enough time for the air between the plates to flow out, and the elastic damping force hinders the motion of the plate. In addition, when the sensitive mass block vibrates up and down, as shown in Figure 1a, the upper and lower surfaces of the mass block are subjected to air friction forces; this is called slide-film air damping. In this case, squeeze-film damping has a far greater effect than slide-film damping, so we only consider the effect of the squeeze-film damping on the dynamic characteristics of the accelerometer.

Suppose that the plate is parallel to the other plate in the *x–y* plane of a Cartesian coordinate system, as shown in Figure 3b, and the dimensions of the plates are much larger than the distance between them. In this case, the effect of squeeze-film air damping can be expressed by the Reynolds equation [46]:

(4) $$\frac{\partial }{\partial x}(\frac{\rho h^{3}}{\mu }\frac{\partial p}{\partial x})+\frac{\partial }{\partial y}(\frac{\rho h^{3}}{\mu }\frac{\partial p}{\partial y})=12\frac{\partial (\rho h)}{\partial t}$$

where ρ is the air density, h is the gap distance between two plates, μ is the viscosity coefficient of air, p is the air pressure between the plates, (x,y) are the space coordinate components, and t is time.

When the movable plate vibrates with the displacement of $h=h_{1}\cos(\omega t)$, the viscous damping force $F_{0}$ and elastic damping force $F_{1}$ on the movable plate can be obtained by solving Equation (4) [47]

(5) $$F_{0}=\frac{64p_{0}l_{p}w_{p}\sigma }{\pi^{6}}\frac{h_{1}}{h_{0}}\lambda_{0}$$

(6) $$F_{1}=\frac{64p_{0}l_{p}w_{p}\sigma^{2}}{\pi^{8}}\frac{h_{1}}{h_{0}}\lambda_{1}$$

where $l_{p}=2b$ is the plate length; $w_{p}=2a$ is the plate width; $\beta =w_{p}/l_{p}$ is the aspect ratio of the plate; $h_{0}$ is the initial gap distance between the plates; $p_{0}$ is the air pressure between the plates under the initial conditions; $\sigma =12\mu \omega l_{p}^{2}/h_{0}^{2}p_{0}$ is the squeeze number; and $\lambda_{0}$ and $\lambda_{1}$ are constants:

(7) $$\lambda_{0}=\underset{m,n;\mathrm{odd}}{\Sigma }\frac{m^{2}+{\left(n/\beta \right)}^{2}}{{\left(mn\right)}^{2}\{{\left[m^{2}+{\left(n/\beta \right)}^{2}\right]}^{2}+\sigma^{2}/\pi^{4}\}}$$

(8) $$\lambda_{1}=\underset{m,n;\mathrm{odd}}{\Sigma }\frac{1}{{\left(mn\right)}^{2}\{{\left[m^{2}+{\left(n/\beta \right)}^{2}\right]}^{2}+\sigma^{2}/\pi^{4}\}}$$

## 3. Structural Design and Optimization

The physical model of the capacitive accelerometer was established by Ansys, and the initial values of the structural parameters are listed in Table 1.

### 3.1. Modal Analysis

The elastic microbeam is a key part of the micro-accelerometer, and is closely related to the sensitivity and range of the sensor. It also has an important impact on dynamic performance.

In the design of the micro-accelerometer, modal analysis, including natural frequencies and mode shapes, should be considered first. According to the analysis results, reasonable size-matching of the elastic beam was obtained, and could be used to widen the gap between the natural frequency in the sensitive direction (the direction of the acceleration to be measured) and that of the non-sensitive direction to improve sensitivity and resolution.

#### 3.1.1. Modal Analysis of the Micro-Accelerometer

**Figure 4:**
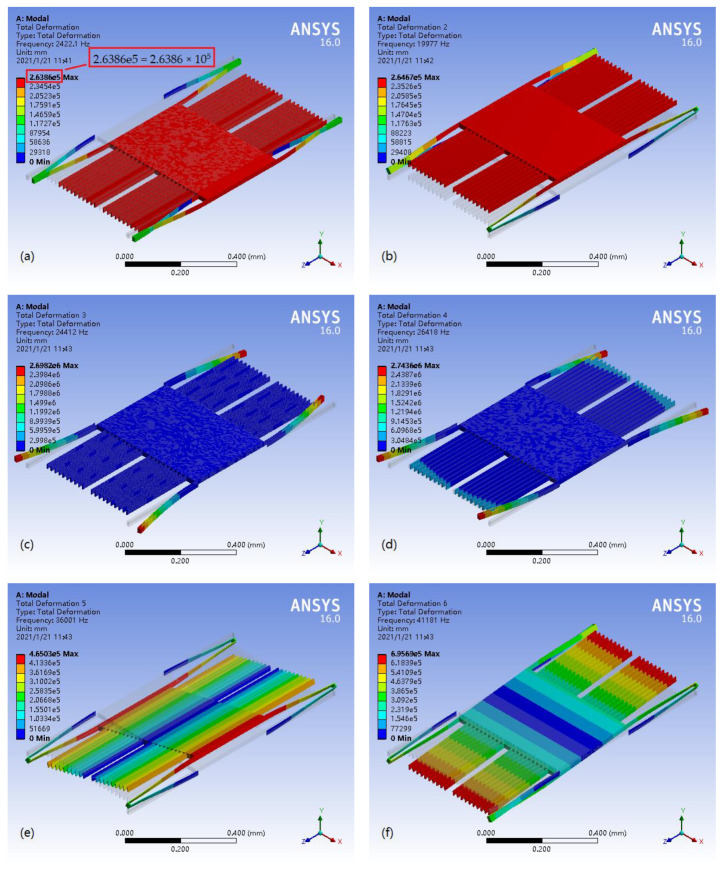
Modal analysis of the capacitive accelerometer based on the SiC microstructure. (**a**) The first mode is the translational motion along the *x*-axis; (**b**) the second mode is the translational motion along the *y*-axis; (**c**) the third mode is the translational motion along the *z*-axis; (**d**) the fourth mode is the rotational motion around the *y*-axis; (**e**) the fifth mode is the rotational motion around the *z*-axis; (**f**) the sixth mode is the rotational motion around the *x*-axis.

In the modal analysis, it was assumed that the density, Young’s modulus, and Poisson’s ratio of silicon carbide were 3200 kg/m^3^, 4.3 × 10^11^ Pa, and 0.17 [48], respectively. With this in mind, a modal analysis of the capacitive micro-accelerometer with folded support beams fixed on the anchors was carried out. The analysis results are shown in Figure 4. The accelerometer has six main modes, including natural frequencies and mode shapes in six degrees of freedom. As can be seen from the mode shape in Figure 4a, the first mode is the translational motion along the *x*-axis, which is sensitive to external acceleration, so it needs to be strengthened. Figure 4b,c shows that the second and third modes are the non-sensitive vibration modes that move along the *y* axis and *z*-axis, respectively, so they need to be suppressed. Similarly, as shown in Figure 4d–f, the fourth, fifth, and sixth modes are the rotational motions around the *y*-axis, *z*-axis, and *x*-axis, respectively, and they also need to be suppressed. The natural frequencies from the first mode to the sixth mode of the accelerometer were 2422.1; 19,977; 24,412; 26,418; 36,001; and 41,181 Hz, respectively.

#### 3.1.2. Optimization of the Structural Parameters

**Figure 5:**
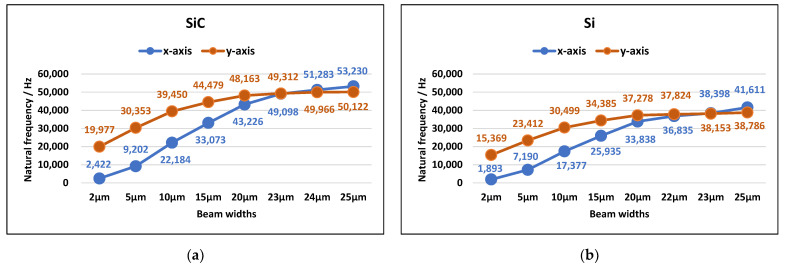
Natural frequencies in the first-two order modes versus the beam width of the capacitive accelerometer based on (**a**) the SiC microstructure and (**b**) the Si microstructure. (The blue line is the natural frequency in the mode moving along the *x*-axis, while the red line is the natural frequency in the mode moving along the *y*-axis.)

Modal analysis of the accelerometer with different widths of the folded support beams was also carried out, and the results showed that the width of the folded beams had an important effect on the natural frequency and mode shape of the accelerometer. As illustrated in Figure 5a, the natural frequency in any order mode of the SiC-based accelerometer increased with an increase in beam width. When the beam-width ranged from 2 to 23 μm, the motion moving along the *x*-axis is always the first-order mode shape, and the motion moving along the *y*-axis is always the second-order. However, when the beam width is more than 23 μm, the order of vibration modes changes; the motion moving along the *y*-axis becomes the first-order mode shape, and the motion moving along the *x*-axis becomes the second-order. At this time, when the accelerometer is excited, it will vibrate along the *y*-axis first. However, the y-axis is not the sensitive direction of the accelerometer, so it is not conducive to the normal measurement of external accelerations.

It can also be seen in Figure 5a that, with an increase in the width of the folded beam, the natural frequency of the first-order mode gradually approaches that of the second-order mode. That is, the thicker the beam width, the more easily cross-coupling occurs. When the accelerometer vibrates along the *x*-axis, an increase in beam width will also cause vibrations along the *y*-axis, and this is not supported in the design of the micro-accelerometer. Therefore, to reduce the cross-coupling of the *x* and *y* axes, the width of the folded beam should be much smaller than 23 μm. According to the above analysis, the width of the beam should be 4 μm, so that the accelerometer achieves the two advantages of a large range and low cross-coupling at the same time.

As shown in Figure 5b, similar results also appeared in the modal analysis of the silicon-based accelerometer with the same structure, in which it was assumed that the density, Young’s modulus, and Poisson’s ratio of silicon in the (111) direction were 2330 kg/m^3^, 1.9 × 10^11^ Pa, and 0.28, respectively [49]. The natural frequency in any order mode increased with an increase in beam width. When the beam width ranged from 2 to 22 μm, the motion moving along the *x*-axis was the first mode. However, if the beam width was more than 22 μm, the motion moving along the *y*-axis became the first mode. The thicker the beam width, the more easily cross-coupling occurred.

#### 3.1.3. Modal Comparison between the SiC-Based Structure and the Si-Based Structure

**Figure 6:**
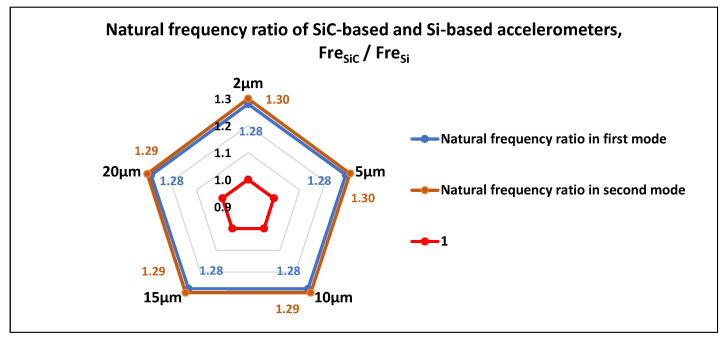
Comparison between the natural frequencies of the SiC-based accelerometer and Si-based accelerometer with the same structure.

We also compared the natural frequencies in the first two modes of the SiC-based accelerometer with those of the Si-based accelerometer with the same structure, as shown in Figure 6. The results show that when the beam width ranged from 2 to 20 μm, the ratios of the first-order natural frequency of the two accelerometers ($Fre_{SiC}/Fre_{Si}$) were always equal to 1.28, and the ratios of the second-order natural frequency were approximately equal to 1.29. Therefore, the SiC-based accelerometer has a higher natural frequency than the silicon(111) accelerometer with the same structure. A high natural frequency not only effectively avoids the resonance of the structure, which may cause structural damage, but also broadens the working frequency range of the accelerometer.

### 3.2. Dynamic Characteristic Analysis

Damping is an important parameter affecting the dynamic performance of the micro-accelerometers. For some accelerometers, the damping characteristics directly determine their working frequency ranges and dynamic accuracies.

#### 3.2.1. Squeeze-Film Air Damping

**Figure 7:**
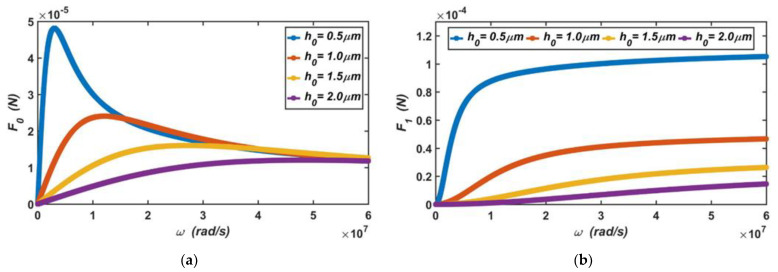
(**a**) The dependence of the viscous damping force on the vibration frequency of the moving plate, and (**b**) the dependence of the elastic damping force on the vibration frequency of the moving plate when the gap between the movable electrode plate and fixed electrode plate ranges from 0.5 to 2 μm.

In the accelerometer, it was assumed that the vibration amplitude of the moving plate was $h_{1}=0.1\;\mu m$. According to Equations (5) and (6), when the gap between the capacitive plates ranges from 0.5 to 2 μm, the dependence of the viscous damping force produced by the air film on the vibration frequency is shown in Figure 7a, and the dependence of the elastic damping force on the vibration frequency is shown in Figure 7b.

It can be seen from the curves in the two figures that when the vibration frequency of the moving plate was low, the viscous damping force of the air film increased linearly with an increase in the vibration frequency, and the elastic damping force increased slowly. When the vibration frequency was higher than a certain value, the viscous damping force of the air film decreased with an increase in the vibration frequency, but the elastic damping force increased rapidly. These results show that when the moving plate vibrates, damping is the main form of air film at a low frequency, and stiffness is the main form of air film at a high frequency. In this capacitive accelerometer, due to its low vibration frequency in operation, viscous damping force is the main source of the damping.

#### 3.2.2. Analysis in the Frequency Domain

The logarithmic amplitude–frequency characteristics of the micro-accelerometer can be expressed as

(9) $$L(\omega )=-20\mathrm{lg}\sqrt{{\left(1-\frac{\omega^{2}}{\omega_{n}^{2}}\right)}^{2}+4\varsigma^{2}\frac{\omega^{2}}{\omega_{n}^{2}}}$$

where ω represents the vibration frequency of the micro-accelerometer, and $\omega_{n}$ represents the natural frequency of the micro-accelerometer. ς is the damping ratio of the system.

**Figure 8:**
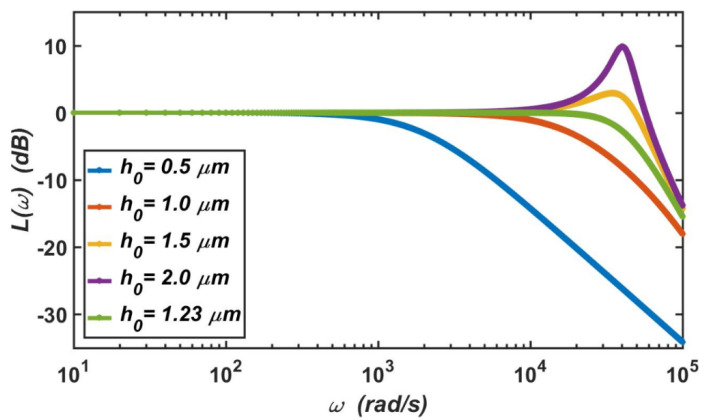
Logarithmic amplitude-frequency characteristics of the micro-accelerometer when the gap between the movable electrode plate and fixed electrode plate ranges from 0.5 to 2 μm.

Figure 8 shows the amplitude–frequency characteristics of the micro-accelerometer at standard atmospheric pressure, which are only determined by the relative damping ratio of the system. It can be seen from the figure that when the gap between the capacitive plates was $h_{0}=0.5\;\mu m$ or $h_{0}=1\;\mu m$, the relative damping ratios were ς = 10.5 and ς = 1.31, respectively, and the system was in the state of overdamping. At this time, the amplitude–frequency curve had no resonant peak, but the working bandwidth was narrow. When the gap between the capacitive plates was $h_{0}=1.5\;\mu m$ or $h_{0}=2\;\mu m$, the relative damping ratios were ς=0.39 and ς=0.16, respectively, and the system was in the state of underdamping. At this time, the bandwidth of the capacitive accelerometer was large. However, when the external vibration frequency approached its natural frequency, the accelerometer will resonate, and its vibration amplitude will become very large. In this case, the system may be damaged; this outcome is not supported in the design of the accelerometer. Therefore, the most appropriate damping ratio of the system is ς≈0.7, at which the micro-accelerometer achieves two advantages at the same time. The first is that it has a large working bandwidth, and the second is that its amplitude-frequency curve has no resonant peak. Based on this, the gap between the capacitive plates should be $h_{0}=1.23\;\mu m$.

#### 3.2.3. Analysis in the Time Domain

**Figure 9:**
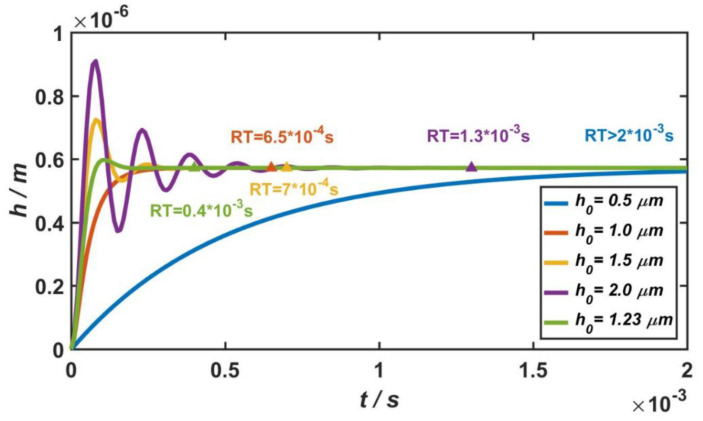
Step responses of the micro-accelerometer when the gap between the movable and fixed electrode plates ranges from 0.5 to 2 μm.

As mentioned in Section 2.2, the micro-accelerometer could be equivalent to a second-order damping system. When the external acceleration was *a* = 100 g, the step response of the micro-accelerometer is shown in Figure 9. If the system is in the state of over-damping, the smaller the damping ratio of the system, the shorter the response time (RT) of the system. As shown by the blue and red curves in Figure 9, when the gap between the capacitive plates was $h_{0}=0.5\;\mu m$ or $h_{0}=1\;\mu m$, the relative damping ratios were ς=10.5 and ς=1.31, and the response times were $RT>2\times 10^{-3}$ s and $RT=6.5\times 10^{-4}$ s, respectively. However, in the case of underdamping, the greater the damping ratio of the system, the shorter the response time. As shown by the yellow and purple curves in Figure 9, when the gap between the capacitive plates was $h_{0}=1.5\;\mu m$ or $h_{0}=2\;\mu m$, the relative damping ratios were ς=0.39 and ς=0.16, and the response times were $RT=7\times 10^{-4}$ s and $RT=1.3\times 10^{-3}$ s, respectively.

In general, when the relative damping ratio of the micro-accelerometer is ς≈0.7, the dynamic characteristics of system are at their best. The system responds quickly, and a stable output value can be achieved in a short period of time. Therefore, in the design of the micro-accelerometer, if there are no other constraints, the damping ratio of the structure should be ς≈0.7 to ensure the optimal dynamic characteristics of the system. In this case, for the capacitive accelerometer in this paper, the gap between the movable and fixed electrode plates should be $h_{0}=1.23\;\mu m$. Based on this, the step response of the micro-accelerometer is shown by the green curve in Figure 9, and the response time is $RT=4\times 10^{-4}$ s.

## 4. Performance Characterization

After the structural parameters of the accelerometer were optimized and determined, its performance characteristics could be obtained through FEM simulations.

### 4.1. Scale Range

**Figure 10:**
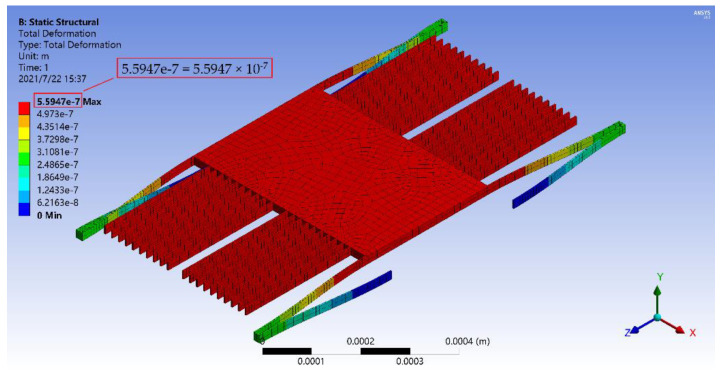
Deformation nephogram of the micro-accelerometer under the acceleration of 100 g along the *x*-axis.

First, mechanical analysis of the accelerometer was carried out at room temperature. Figure 10 shows the total deformation of the accelerometer under the acceleration of 100 g along the *x*-axis. The final displacement of the mass block with the moving plates was 0.559 μm, which was less than the gap distance between the moving and fixed electrode plates ($h_{0}=1.23\;\mu m$), so the accelerometer will not be damaged during operation.

**Figure 11:**
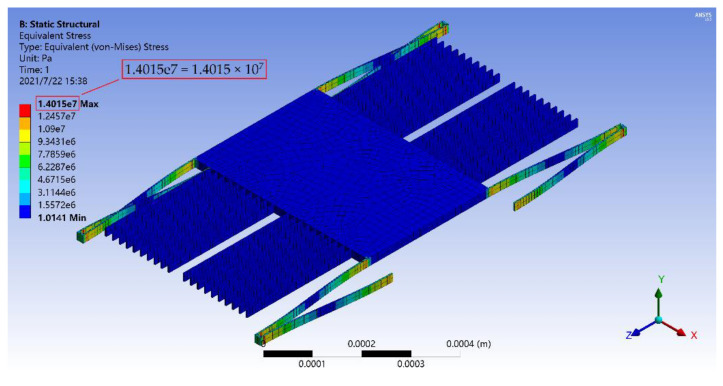
Stress nephogram of the micro-accelerometer under the acceleration of 100 g along the *x*-axis.

Then, structural strength analysis of the accelerometer was carried out at room temperature. Figure 11 shows the stress distribution of the accelerometer under the acceleration of 100 g along the *x*-axis. It can be seen that large stresses existed at the connection between the folded beam and the mass block, the connection between the folded beam and the anchor and the corner of the folded beam, but its maximum value (14.015 MPa) was much less than the flexural strength of silicon carbide (359 MPa). Therefore, under a large acceleration, the structure could still maintain a good shape without fracturing.

Based on the above analysis, the accelerometer can effectively meet the measurement requirements of the acceleration range from 0 to 100 g. In addition, the silicon(111) accelerometer with the same structure was also simulated, and its scale range was 0 to 61 g. Therefore, the range of the SiC-based accelerometer was 1.64 times of that of the silicon(111) with the same structure.

### 4.2. Sensitivity

**Figure 12:**
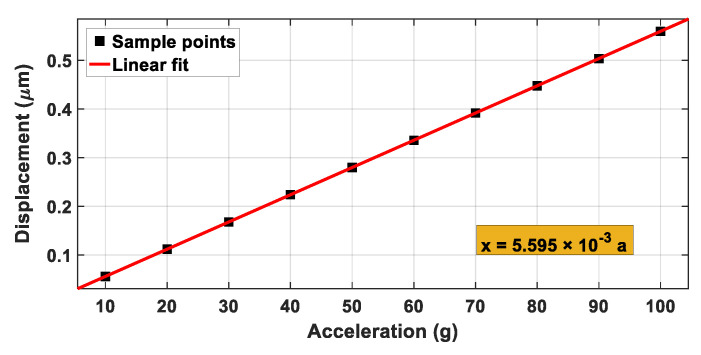
Relationship between displacement and acceleration.

The mechanical sensitivity of the accelerometer is one of the important indexes that which represents the corresponding displacement generated by the acceleration per unit. Ten groups of simulation experiments under different accelerations were made at room temperature, and the experiment results are shown in Table 2. The relationship between the displacement and the acceleration was plotted as Figure 12, and it could be derived from linear regression as follows:

(10) x=0.005595⋅a

Therefore, the sensitivity of the accelerometer (S=x/a) was 0.0056 μm/g.

### 4.3. High-Temperature Performances

Silicon carbide is an attractive material for high-temperature semiconductor devices, so dozens of FEM simulations, including mechanical analysis and structural strength analysis, were made to get the thermal performances of the accelerometer at high temperatures. The material properties of silicon carbide at different temperatures are shown in Table 3 [50], and it can be seen from it that the density, elastic modulus, and Poisson’s ratio of silicon carbide decrease with the increase in temperature. Therefore, the mass, the stiffness coefficient, and other parameters of the accelerometer will change accordingly.

**Figure 13:**
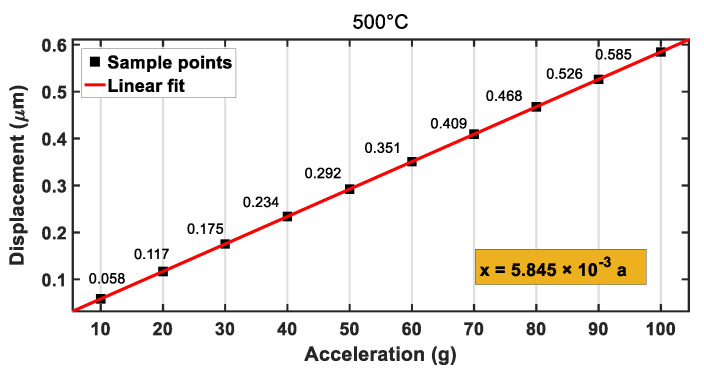
Relationship between displacement and acceleration at 500 °C.

**Figure 14:**
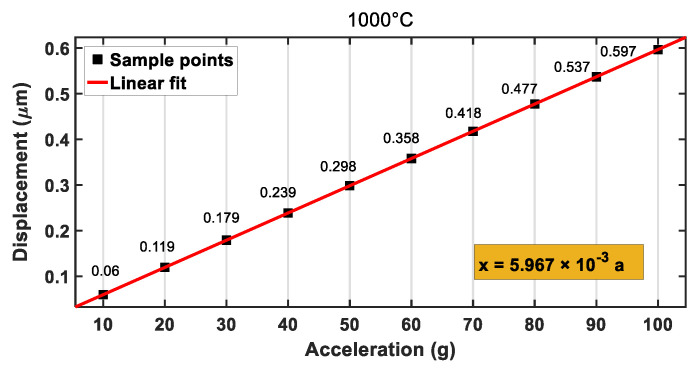
Relationship between displacement and acceleration at 1000 °C.

**Figure 15:**
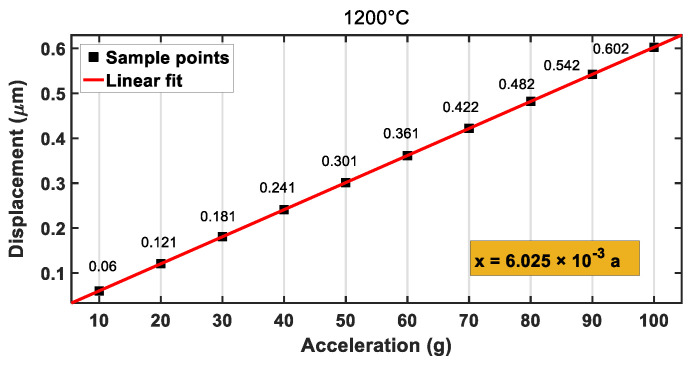
Relationship between displacement and acceleration at 1200 °C.

In the mechanical analysis, characteristic parameters of silicon carbide at different temperatures (see Table 3) were set in Ansys. Then, the temperature of the accelerometer was set to the corresponding value (500, 1000, 1200 °C). In the case, the dependences of the displacements of the accelerometer on the external accelerations at 500, 1000, and 1200 °C are shown in Figure 13, Figure 14 and Figure 15, respectively. When the temperature was increased to 500, 1000, or 1200 °C, the final deformations of the accelerometer were 0.585, 0.597, and 0.602 μm, respectively, under the external acceleration of *a* = 100 g, which was less than the gap distance between the moving and fixed electrode plates ($h_{0}=1.23\;\mu m$). After the linear fitting of the sample points, the mechanical sensitivities of the accelerometer were 0.0058, 0.0059, and 0.0060 μm/g, respectively. By comparing the sensitivities of the accelerometer at different temperatures, it was found that the sensitivities increased with the increase in temperature, but the change was not very notable, which was conducive to the measurement of the accelerometer at high temperatures. Therefore, the SiC-based accelerometer had the advantage of low temperature sensitivity, which leads to high reliability.

**Figure 16:**
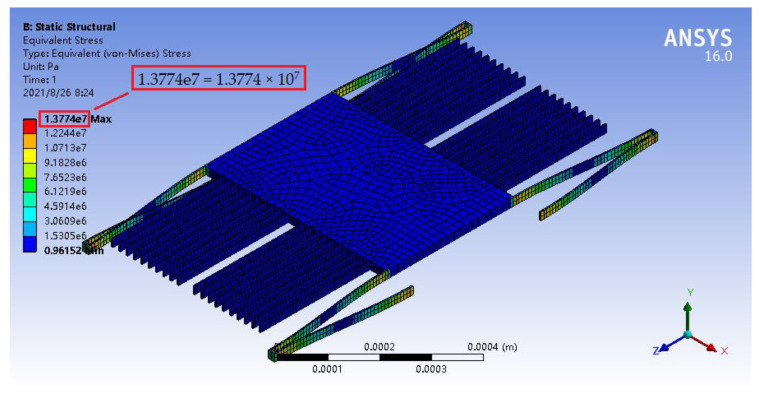
Stress nephogram of the micro-accelerometer under the acceleration of 100 g at 500 °C.

**Figure 17:**
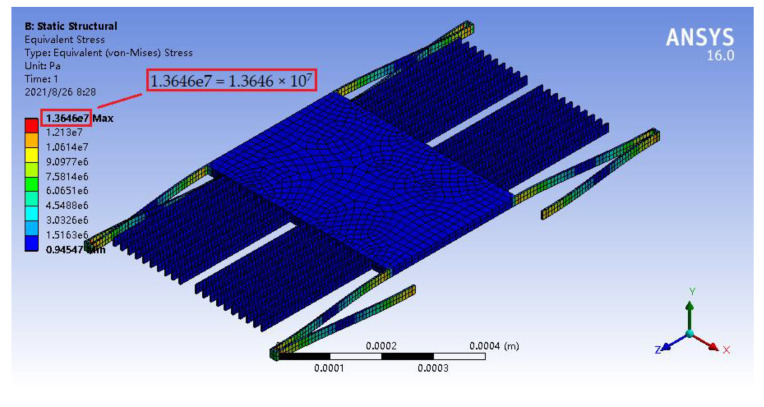
Stress nephogram of the micro-accelerometer under the acceleration of 100 g at 1000 °C.

**Figure 18:**
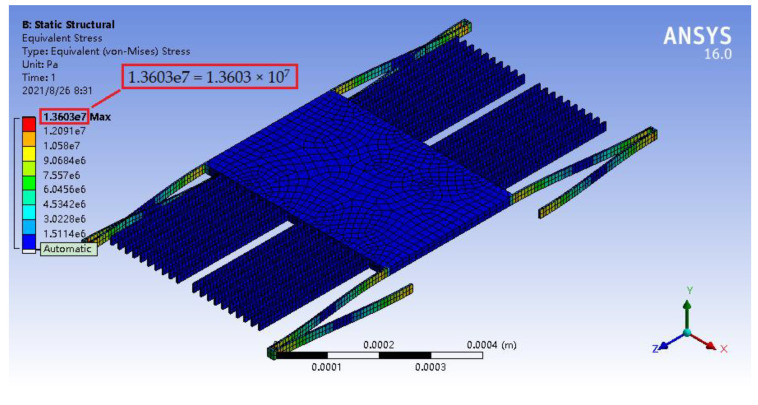
Stress nephogram of the micro-accelerometer under the acceleration of 100 g at 1200 °C.

In the structural strength analysis, its parameter setting and temperature setting were similar to those in the mechanical analysis. In the case, when the external acceleration was *a* = 100 g, the maximum stresses of the accelerometer at 500, 1000, and 1200 °C were 13.774, 13.646, and 13.603 MPa, respectively, as illustrated in Figure 16, Figure 17 and Figure 18, which were far less than the flexural strengths of silicon carbide at high temperatures (see Table 3).

Therefore, the accelerometer could still effectively meet the measurement requirements of the acceleration range from 0 to 100 g at high temperatures.

## 5. Discussion

As could be seen from this article, the process flow and the working principle of the capacitive accelerometer based on a silicon carbide microstructure was introduced. Through the modal analysis of the micro-accelerometer, the structural parameters of the elastic microbeam were optimized. This not only placed the sensitive mode in the first vibration mode, but also widened the frequency difference between the first vibration mode and other vibration modes to avoid cross-coupling. It was found that the ratio of the natural frequency in each mode of the SiC-based accelerometer, compared to that of a silicon(111) accelerometer with the same structure, was approximately equal to 1.3, so the SiC-based accelerometer has a higher natural frequency. This means it can not only effectively avoid the resonance of the structure, which may cause structural damage, but also broaden the working frequency range of the accelerometer.

It is well known that squeeze-film air damping is produced between the moving and fixed electrode plates when the micro-accelerometer vibrates. We found that when the vibration frequency was low, the viscous damping force of the air film increased linearly with the increase in vibration frequency, and the elastic damping force increased slowly. When the vibration frequency was high, the viscous damping force of the air film decreased with the increase in vibration frequency, but the elastic damping force increased rapidly.

The second-order linear system of the accelerometer was analyzed in the frequency domain, and its amplitude-frequency characteristics showed that the accelerometer obtained two simultaneous advantages when the relative damping ratio is 0.7. One is a large working bandwidth, and the other is an amplitude-frequency curve that had no resonant peak. In this case, the gap between capacitive plates should be 1.23 μm. The second-order system of the accelerometer was also analyzed in the time domain, and its step response showed that, when the damping ratio of the micro-accelerometer was 0.7, the system responded quickly, and a stable output value could be achieved in a short period of time. In this case, when the external acceleration was *a* = 100 g, the response time of the accelerometer was 0.4 ms.

The range of this accelerometer was from 0 to 100 g, and the range of a silicon(111) accelerometer with the same structure was from 0 to 61 g. Therefore, the range of the SiC-based accelerometer is 1.64 times of that of silicon(111) accelerometer. When the temperature was increased to 500, 1000, or 1200 °C, the mechanical sensitivities of the accelerometer were 0.0058, 0.0059, and 0.0060 μm/g, respectively, and it could be seen from it that the sensitivities increased with the increase in temperature, but the change was not very notable. Therefore, the SiC-based accelerometer had the advantage of low temperature sensitivity, which leads to high reliability of the device. It is worth noting that, although the theory presented in this study did not account for all the ground truth of silicon and silicon carbide, the results provided great potential for the application of SiC-based accelerometers.

## 6. Conclusions

In this study, a capacitive accelerometer based on a silicon carbide microstructure was designed and investigated by FEM. It had many advantages, such as low cross-coupling and good dynamic characteristics. Compared to a silicon(111) accelerometer with the same structure, it had a higher natural frequency, a wider range, and a higher working temperature of up to 1200 °C. Therefore, this research effectively validated the advantages of a capacitive accelerometer based on the silicon carbide microstructure over conventional silicon devices in the field of MEMS inertia measurement.

## Figures and Tables

**Table 1:** The initial values of the structural parameters for the capacitive accelerometer.

Accelerometer	Length (μm)	Width (μm)	Thickness (μm)
Mass block	500	350	20
Movable electrode plates	300	4	20
Fixed electrode plates	300	4	20
Folded support beams	350	2	20

**Table 2:** Displacements of the micro-accelerometer under different accelerations along the x-axis.

External Acceleration	Displacement
10 g	5.5947 × 10^−2^ μm
20 g	1.1189 × 10^−1^ μm
30 g	1.6784 × 10^−1^ μm
40 g	2.2379 × 10^−1^ μm
50 g	2.7973 × 10^−1^ μm
60 g	3.3568 × 10^−1^ μm
70 g	3.9163 × 10^−1^ μm
80 g	4.4757 × 10^−1^ μm
90 g	5.0352 × 10^−1^ μm
100 g	5.5947 × 10^−1^ μm

**Table 3:** Characteristic parameters of silicon carbide at different temperatures.

Property	500 °C	1000 °C	1200 °C
Density (g/cm^3^)	3.14	3.11	3.10
Elastic modulus (GPa)	404	392	387
Poisson’s ratio	0.159	0.157	0.157
Flexural strength (MPa)	359	397	437
Thermal expansion coefficient (10^−6^ K^−1^)	4.4	5	5.2

## Data Availability

Not applicable.
